# Supplementary material for: Viremia Kinetics in Pigs Inoculated with Modified Live African Swine Fever Viruses
Source: Vaccines (Basel). 2025 Jun 26;13(7):686. doi: 10.3390/vaccines13070686 (PMC12298559; doi:10.3390/vaccines13070686)
Supplement: Supplementary file 1 [file vaccines-13-00686-s001.zip › vaccines-3675357-supplementary.pdf]

Table S1A. Viremia in pigs after inoculation with ASF MLV and challenge: ASF MLV with a Single Deletion.

Virus strain	Doses of inoculation	Clinical signs pi	Term/ viraemia dpi	Challenge dose/strain	Clinical signs pc	Term/ viraemia dpc	Survival rate	Persisten.	Ref
ASFV-G-ΔH108R	10^2 HAD_{50}	—/+	4-21/2-8<br>28/4-7	10^2 HAD_{50}<br>ASFV-G	—/+	4-14/4-6<br>21/2-4	4/5	N	[37]
SY18ΔI226R*	10^4 $TCID_{50}$	—	3-19/3-7<br>21/4-6	$10^{2.5}$ $TCID_{50}$<br>ASFV SY18	—/+	2-24/3-7<br>26/3-6	5/5	ND	[38]
	10^7 $TCID_{50}$	—	3-19/4-7<br>21/4-6	10^4 $TCID_{50}$<br>ASFV SY18	—	2-24/3-6<br>26/ND	5/5	ND	
ASFV-G-9GL	10^2 HAD_{50}	—	4-14/ND-7<br>21/ND-6	10^3 HAD_{50}<br>ASFV-G	+	4-14/4-8	2/5	N	[43]
	10^2 HAD_{50}	—	7-21/2-7<br>28/3-6		+/—	4-14/2-5<br>21/ND-7	5/5	N	
	10^3 HAD_{50}	—	4-14/2-6<br>21/3-6		—/+	4-14/2-5<br>21/2-3	5/5	N	
	10^3 HAD_{50}	—	4-21/2-7<br>28/2-6		—	4-11/2-7<br>21/ND-6	5/5	N	
	10^4 HAD_{50}	+	4-7/2-3		**N**	N	0/5	N	
ASFV-G-ΔA137R**	10^2 HAD_{50}	—	4-21/2-6<br>28/5	10^2 HAD_{50}<br>ASFV-G	—	4-14/2-5<br>21/2-3	5/5	ND-15	[44]
ASFV-Georgia-ΔI177L	10^2 HAD_{50}	—	4/ND<br>7-21/ND-7<br>28/ND-6	10^2 HAD_{50}<br>ASFV-G	—	4-14/ND-6<br>21/ND	5/5	+	[39]
	10^4 HAD_{50}	—	4-21/2-7<br>28/3-5		—	4-14/ND-6<br>21/ND	5/5	+	
	10^6 HAD_{50}	—	4-21/ND-8<br>28/ND-6		—	4-14/ND-6<br>21/ND	5/5	+	
ASFV-G-ΔI177L	10^2 HAD_{50}	—	4/ND-3<br>7-21/ND-7<br>28/ND-6	10^2 HAD_{50}<br>ASFV-G	—	4-14/ND-6<br>21/ND-3	5/5	N	[42]
ASFV-G-ΔI177 (ON)	2×10^6 HAD_{50}	—	4-21/ND-4<br>28/ND-3		—	4-21/ND-3	5/5	N	
ASFV-GZΔI73R*	10^3 $TCID_{50}$	—	14/ND-4<br>21-28/ND	10^4 $TCID_{50}$<br>ASFV-GZ	—/+	5-14/ND-6<br>21/ND-3	3/3	ND	[46]
	10^5 $TCID_{50}$	—	0-7/ND<br>9-14/ND-4<br>21-28/ND	N	**N**	N	3/3	ND	
		—	7-14/3-5<br>21/3-4<br>28/ND-3<br>35-56/ND	N	N	N	5/5	—/+	

ND — no detection
N — data not shown
ON — oronasal inoculation
* - DNA copies/mL (log_{10})
** - Ct value

Green shading corresponds to the absence of clinical signs (-) after inoculation with ASF MLV or challenge with highly virulent ASFV, absence of infectious virus or ASFV DNA in blood samples at the day of challenge, 100% survival, absence of infectious virus or ASFV DNA in organ or tissue samples at the end of the experiment.

Yellow shading corresponds to ASF clinical signs scores after inoculation with ASF MLV or challenge with highly virulent ASFV ≤ 6 points, presence of infectious virus (≤ 10^4 $HAD_{50}/TCID_{50}$) or ASFV DNA (Ct ≥ 35) in blood samples on the day of challenge, survival ≥ 80%, presence of infectious virus (≤ 10^4 $HAD_{50}/TCID_{50}$) or ASFV DNA (Ct ≥ 35) in organ or tissue samples at the end of the experiment.

Red shading corresponds to ASF clinical signs scores after inoculation with ASF MLV or challenge with highly virulent ASFV > 6 points, presence of infectious virus (> 10^4 $HAD_{50}/TCID_{50}$) or ASFV DNA (Ct < 35) in blood samples on the day of challenge, < 80% survival, presence of infectious virus (> 10^4 $HAD_{50}/TCID_{50}$) or ASFV DNA (Ct < 35) in organ or tissue samples at the end of the experiment.

Table S1B. Viremia in pigs after inoculation with ASF MLV and challenge: ASF MLV with Two and more Deletions.

Virus strain	Doses of inoculation	Clinical signs pi	Term/ viraemia dpi	Challenge dose/strain	Clinical signs pc	Term/ viraemia dpc	Survival rate	Persisten.	Ref
ASFV-G-ΔMGF	10^2 HAD_{50}	—	4-21/2-5<br>28/ND-3	10^3 HAD_{50}<br>ASFV-G	—	4-21/2-5	10/10	N	[47]
	10^4 HAD_{50}	—	4-21/ND-6<br>28/ND-4		—	4-21/2-6	10/10	N	
ASFV-ΔH240R-Δ7R	10^3 HAD_{50}	—	1-4/ND<br>7-21/ND-4<br>28/ND-2	$10^{2.5}$ HAD_{50}<br>ASFV<br>HLJ/18	—/+	1/ND-1<br>4-16/1-6<br>21/2-4	5/5	1-4	[48]
	10^5 HAD_{50}	—	1-4/ND<br>7-21/1-2<br>28/ND		—	1-16/1-2<br>21/2-3	5/5	ND-3	
ASFV-MEC-01**	10^5 HAD_{50}	—	7-21/ND-35<br>28/ND	10^2 HAD_{50}<br>Korea/Wild boar/ Hwacheon/2020	—	14-21/ND-30	3/3	+	[49]
	10^5 HAD_{50}	—	7-28/ND-28<br>35/ND		—	7-14/35-15	4/4	+	
ASFV-G-Δ9GL/ΔUK	10^2 HAD_{50}	—	4/ND<br>7-21/ND-7<br>28/ND-5	10^3 HAD_{50}<br>ASFV-G	+/—	4-7/3-8<br>11/3-4<br>14-21/ND-4	4/9	N	[28]
	10^4 HAD_{50}	—	4/ND<br>7-21/4-7<br>28/ND-5		—	4-7/3-8<br>11-14/ND-5-<br>21/ND-3	10/10	N	
	10^6 HAD_{50}	—	4/ND-4<br>7-21/ND-6<br>28/ND-5		+/—	4-7/ND-5<br>11-14/ND-4<br>21/ND-3	14/15	N	
ASFV-G-Δ9GL/ΔUKp10	10^4 HAD_{50}	—	4/ND<br>7-21/2-6<br>28/3-5	10^2 HAD_{50}<br>ASFV-G	—/+	4-14/3-5<br>21/ ND -3	3/5	ND	[50]

Table S1C. Viremia in pigs after inoculation with ASF MLV and challenge: ASF MLV with Two Deletions, Including EP402R.

Virus strain	Doses of inoculation	Clinical signs pi	Term/ viraemia dpi	Challenge dose/strain	Clinical signs pc	Term/ viraemia dpc	Survival rate	Persisten.	Ref
ASFV-SY18-ΔCD2v/UK**	10^4 $TCID_{50}$	—	21-28/ND	10^4 $TCID_{50}$<br>ASFV-SY18	—	3-15/ND<br>18-21/36-37	5/5	37-39	[59]
SY18ΔL60LΔCD2v*	10^5 $TCID_{50}$	—	3/ND<br>7/ND-1<br>14-28/ND	10^2 $TCID_{50}$<br>SY18	—	3/ND<br>7-21/ND-2	5/5	ND-3	[60]
Arm-ΔCD2v-ΔA238L**	10^2 $TCID_{50}$	—	0/38-39<br>3-5/ND-37<br>7-14/ND-35<br>21-28/ND	10^2 HAD_{50}<br>ASF/Korea/<br>Pig/Paju/2019	—	3-14/ND-35<br>21/ND-36	4/4	ND-24	[62]
ASFV-G-ΔI177L/ΔEP402R	10^2 $TCID_{50}$<br>(HAD_{50})	—	0-28/ND	10^2 HAD_{50}<br>ASFV-G	—	4/ND<br>7-14/ND-6<br>21/ND-7	5/5	N	[64]
	10^6 $TCID_{50}$<br>(HAD_{50})	—	0-28/ND		—	4/ND<br>7-14/ND-6<br>21/ND-5	5/5	N	
BeninΔDP148RΔEP402R	10^3 $TCID_{50}$	—/+	3/ND<br>5-14/ND-6<br>20-42/ND	10^4 HAD_{50}<br>Benin 97/1	—	3-20/ND	3/3	N	[66]
BeninΔDP148RΔEP153RΔ EP402R	10^4 $TCID_{50}$	—	0-44/ND	10^3 HAD_{50}<br>Benin 97/1	+	3-13/ND-6<br>20/ND-5	6/8	N	
BeninΔDP148RΔEP153R	10^5 $TCID_{50}$	+	0/ND<br>3-28/4-7<br>39/ ND-2	10^3 HAD_{50}<br>Benin 97/1	—	7/ND<br>18/ND-4	6/6	N	
BeninΔDP148R	10^5 $TCID_{50}$	+	0/ND<br>5-28/4-6<br>35/0-4<br>40/ND	10^4 HAD_{50}<br>Benin 97/1	—	7/ ND<br>18/ND-2	5/5	N	
ASFV-G-Δ9GL	10^3 $TCID_{50}$	—	4-35/4-5	10^3 $TCID_{50}$<br>ASFV-G	—	4-14/3-4<br>21/ND	4/4	N	[67]
ASFV-G-Δ9GL/ΔCD2v	10^3 $TCID_{50}$	—	4-35/ND		+	4/6	0/4	N	
ASFV-G-Δ9GL/ ΔCD2v/ΔEP153R	10^3 $TCID_{50}$	—	4-35/ND		+	4/6	0/4	N	
SY18ΔMGF/CD2v*	10^4 $TCID_{50}$	—	3-21/ND	$10^{2.5}$ $TCID_{50}$	+	2-24/3-8	3/5	3-9	[38]

				ASFV SY18		26/ND			

Table S1D. Viremia in pigs after inoculation with ASF MLV and challenge: Potential ASF MLV candidates.

Virus strain	Doses of inoculation	Clinical signs pi	Term/ viraemia dpi	Challenge dose/strain	Clinical signs pc	Term/ viraemia dpc	Survival rate	Persisten.	Ref
VNUA-ASFV-LAVL2	10^2 HAD_{50}	—	3/ND<br>5-24/2-4<br>28/ND	8 x10^2 HAD_{50}<br>VNUA-<br>ASFV-05L1	—	3/ ND<br>5/ND-2<br>7-28/ND	5/5	ND	[68]
	10^3 HAD_{50}	—	3/ND-2<br>5-24/2-4<br>28/ND-1		—	3/ ND<br>5/ND-2<br>7-28/ND	5/5	ND	
	10^4 HAD_{50}	—	3/2-3<br>5-24/2-4<br>28/ND-2		—	3-28/ND	5/5	ND	
	10^5 HAD_{50}	—	3/3<br>5-24/2-5<br>28/2-3		—	3/2<br>5-28/ND	5/5	ND	
VNUA-ASFV-LAVL3**	10^3 $TCID_{50}$	—	3/ND<br>5-17/35-38<br>21-28/ND	10^3 HAD_{50}<br>VNUA-<br>ASFV-05L1	—	3-5/ND<br>7/ND-37<br>9-28/ND	5/5	ND	[69]
	10^4 $TCID_{50}$	—	3/ND<br>5-21/35-39<br>24-28/ND		—	3-28/ND	5/5	ND	
	10^5 $TCID_{50}$	—	3-21/30-39<br>24-28/ND		—	3-28/ND	5/5	ND	
ΔMGF360/505_Stav*	10^4 HAD_{50}	—	3-7/2-6<br>14-21/ND	10^4 HAD_{50}<br>Stavropol_01/<br>08	—	3-30/ND	5/5	ND	[70]
ASFV-G-ΔMGF*	10^4 HAD_{50}	—	7/ ND-1<br>21/ ND-2<br>28-42/ ND	10^4 HAD_{50}<br>ASFV-<br>Armenia<br>2008	—	4/ ND-1<br>10-14/ ND-2<br>21/ ND	5/5	ND-2	[71]
	10^3 HAD_{50}	—	7-14/ ND-1<br>21-42/ ND		—	4-21/ ND	5/5	ND	
